# Motion analysis for better understanding of psychomotor skills in laparoscopy: objective assessment-based simulation training using animal organs

**DOI:** 10.1007/s00464-020-07940-7

**Published:** 2020-09-09

**Authors:** Koki Ebina, Takashige Abe, Madoka Higuchi, Jun Furumido, Naoya Iwahara, Masafumi Kon, Kiyohiko Hotta, Shunsuke Komizunai, Yo Kurashima, Hiroshi Kikuchi, Ryuji Matsumoto, Takahiro Osawa, Sachiyo Murai, Teppei Tsujita, Kazuya Sase, Xiaoshuai Chen, Atsushi Konno, Nobuo Shinohara

**Affiliations:** 1 Department of Urology, Hokkaido University Graduate School of Medicine, North-15, West-7, North Ward, Sapporo, 060-8638 Japan; 2 Graduate School of Information Science and Technology, Hokkaido University, Sapporo, Japan; 3 Hokkaido University Clinical Simulation Center, Hokkaido University Graduate School of Medicine, Sapporo, Japan; 4 Department of Mechanical Engineering, National Defense Academy, Yokosuka, 239-8686 Japan; 5 Department of Mechanical Engineering and Intelligent Systems, Tohoku Gakuin University, Tagajo, 985-8537 Japan; 6 Graduate School of Science and Technology, Hirosaki University, Hirosaki, 036-8561 Japan

**Keywords:** Laparoscopic surgery, Simulation training, Motion capture, Surgical education

## Abstract

**Background:**

Our aim was to characterize the motions of multiple laparoscopic surgical instruments among participants with different levels of surgical experience in a series of wet-lab training drills, in which participants need to perform a range of surgical procedures including grasping tissue, tissue traction and dissection, applying a Hem-o-lok clip, and suturing/knotting, and digitize the level of surgical competency.

**Methods:**

Participants performed tissue dissection around the aorta, dividing encountered vessels after applying a Hem-o-lok (Task 1), and renal parenchymal closure (Task 2: suturing, Task 3: suturing and knot-tying), using swine cadaveric organs placed in a box trainer under a motion capture (Mocap) system. Motion-related metrics were compared according to participants’ level of surgical experience (experts: 50 ≤ laparoscopic surgeries, intermediates: 10–49, novices: 0–9), using the Kruskal–Wallis test, and significant metrics were subjected to principal component analysis (PCA).

**Results:**

A total of 15 experts, 12 intermediates, and 18 novices participated in the training. In Task 1, a shorter path length and faster velocity/acceleration/jerk were observed using both scissors and a Hem-o-lok applier in the experts, and Hem-o-lok-related metrics markedly contributed to the 1st principal component on PCA analysis, followed by scissors-related metrics. Higher-level skills including a shorter path length and faster velocity were observed in both hands of the experts also in tasks 2 and 3. Sub-analysis showed that, in experts with 100 ≤  cases, scissors moved more frequently in the “close zone (0  ≤ to < 2.0 cm from aorta)” than those with 50–99 cases.

**Conclusion:**

Our novel Mocap system recognized significant differences in several metrics in multiple instruments according to the level of surgical experience. “Applying a Hem-o-lok clip on a pedicle” strongly reflected the level of surgical experience, and zone-metrics may be a promising tool to assess surgical expertise. Our next challenge is to give completely objective feedback to trainees on-site in the wet-lab.

**Electronic supplementary material:**

The online version of this article (10.1007/s00464-020-07940-7) contains supplementary material, which is available to authorized users.

The recent rapid spread of minimally invasive surgery, such as laparoscopic surgery and robotic surgery, that requires specific psychomotor skills has simultaneously brought a new educational method of simulation training outside the operating theater to a wide range of surgical disciplines, including gastrointestinal, gynecological, and urological surgery. On considering both working-hour restrictions and ethical considerations regarding patient safety, educators need to develop efficient training programs, in which assessment of the surgical skill level and valuable feedback are essential. Since June 2017, aiming to develop an effective and low-cost wet-lab model for training in essential laparoscopic surgical skills, we started simulation training using cadaveric porcine organs, including tissue dissection around the aorta, applying a Hem-o-lok in the vascular pedicle, and renal parenchymal closure. In order to complete each training task, participants need to employ various surgical skills using a range of laparoscopic surgical instruments, and we previously reported good construct validity of our cadaveric porcine organ model [1].

There is a growing body of literature on motion analyses of psychomotor skills in laparoscopic surgery, using different types of measurement methods such as an electromagnetic tracking system (e.g., The Imperial College Surgical Assessment Device, ICSAD [2, 3]), optical scale sensors and micro-encoders (e.g., Hiroshima University Endoscopic Surgical Assessment Device, HUESAD [4]), optical sensors (e.g., TrEndo [5, 6]), a computer-software tracking system based on endoscopic video analysis (e.g., Endoscopic Video Analysis, EVA [7]), and infrared camera motion tracking (e.g., iSurgeon [8]). Intracorporeal suturing/knot tying were extensively analyzed in a dry box training environment using non-biological materials, and a shorter task time, shorter path length, faster velocity, and better motion smoothness were previously reported [5, 6, 8–15]. However, to our knowledge, other core surgical skills, such as tissue dissection or applying a vascular clip on a pedicle, have not been fully analyzed.

In the present study, we performed motion capture (Mocap) analysis of multiple surgical instruments among participants with different levels of experience of laparoscopic surgery in a series of wet-lab training sessions using our cadaveric porcine organ model. As abovementioned, because we had already confirmed good construct validity of the present training tasks in our previous study based on experts’ video reviews, we consider that our model is appropriate to advance understanding of the components of surgical dexterity among several core surgical skills in laparoscopy. As described later, our Mocap system can recognize each instrument individually irrespective of instrument exchanges, which enables us to characterize the motions of multiple surgical instruments simultaneously in complex training tasks that require a range of surgical techniques, such as grasping tissue, tissue traction and dissection, applying a Hem-o-lok clip, and suturing/knotting. Our aims were to clarify the motion characteristics according to surgical experiences in a series of wet-lab training sessions, and digitize the level of surgical competency, which facilitates clear feedback of motion parameters to trainees.

## Materials and methods

**Fig. 1:**
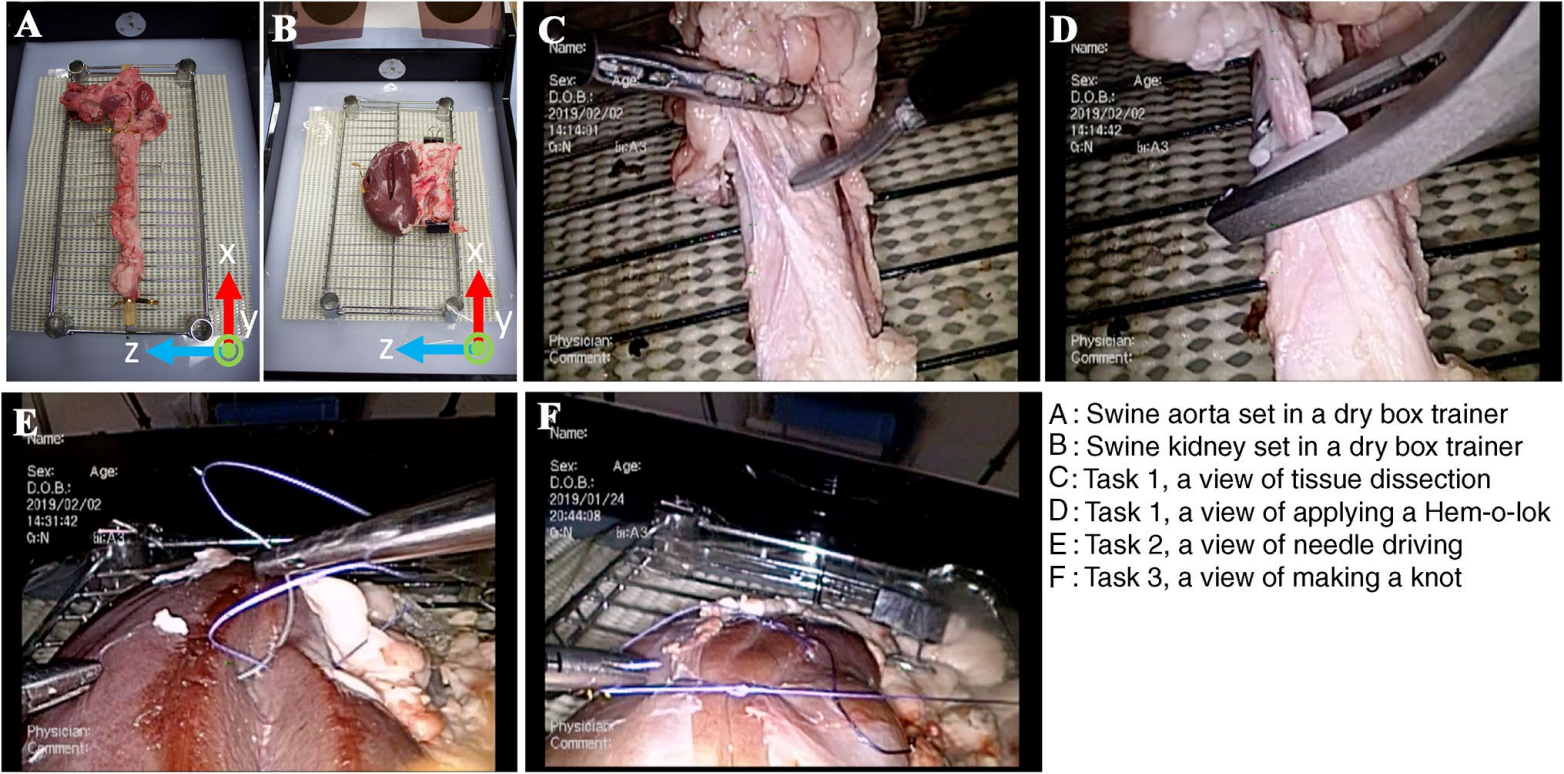
Photographs of the swine organ training model. **A** Swine aorta set in a dry box trainer. **B** Swine kidney set in a dry box trainer. **C** Task 1, a view of tissue dissection. **D** Task 1, a view of applying a Hem-o-lok. **E** Task 2, a view of needle driving. **F** Task 3, a view of making a knot

The institutional review board approved the present study (No. 018-0257). As described above, we previously reported our wet-lab training using cadaveric porcine organs. Briefly, participants performed three tasks: Task 1: tissue dissection around the aorta, dividing encountered mesenteric vessels after applying a Hem-o-lok, Task 2: tissue dissection and division of the renal artery, and Task 3: renal parenchymal closure. We observed good construct validity based on Global Operative Assessment of Laparoscopic Skills (GOALS) and our original assessment sheet, by two blinded experts’ video reviews of all three tasks [1]. We used Task 1 and a modified Task 3 for the present Mocap analysis. Forty-five subjects voluntarily participated in the training. Written informed consent was obtained regarding the use of their data for research. The details of the present training tasks are described in the next paragraph. In all tasks, porcine cadaveric organs were placed in a box trainer (Endowork Pro®, Kyoto Kagaku, Japan, Fig. 1A, B). Porcine organs were purchased from a commercial vendor. Before the training, each task was explained by one of the authors (KE) using recorded movies. During the training, one of the four authors (TA, MH, JF, and NI) was a scopist, using a video system (VISERA Pro Video System Center OTV-S7Pro, Olympus, Japan, Fig. 1A) and zero-degree lens. If participants had problems with simulation, especially medical students, each step of the training task was verbally guided by the scopist. After the training session, completed questionnaires were collected, including demographic data and experience of laparoscopic surgeries. In Japan, the Endoscopic Surgical Skill Qualification (ESSQ) system was initiated in 2004, in which two double-blinded referees evaluate an unedited surgical movie [16, 17], and this certification status was also ascertained. All training sessions were video-recorded, and the subjective mental workload was assessed by NASA Task Load Index after each training session for subsequent analysis.

## The training tasks of the present study

### Task 1

Participants are required to remove the tissues around the aorta, dividing encountered mesenteric vessels after applying a Hem-o-lok (Fig. 1C, D). Usually, 5–7 mesenteric vessels were divided during the task.

### Tasks 2 and 3

Participants are given a 15-cm 2-0 CT-1 VICRYL® thread, and are required to pass the needle from right to left through the kidney parenchyma at three different sites on a kidney (Task 2, Fig. 1E). In Task 3, participants are asked to complete three-square single-throw knots at two different sites on a kidney (Fig. 1F).

## Motion capture analysis

**Fig. 2:**
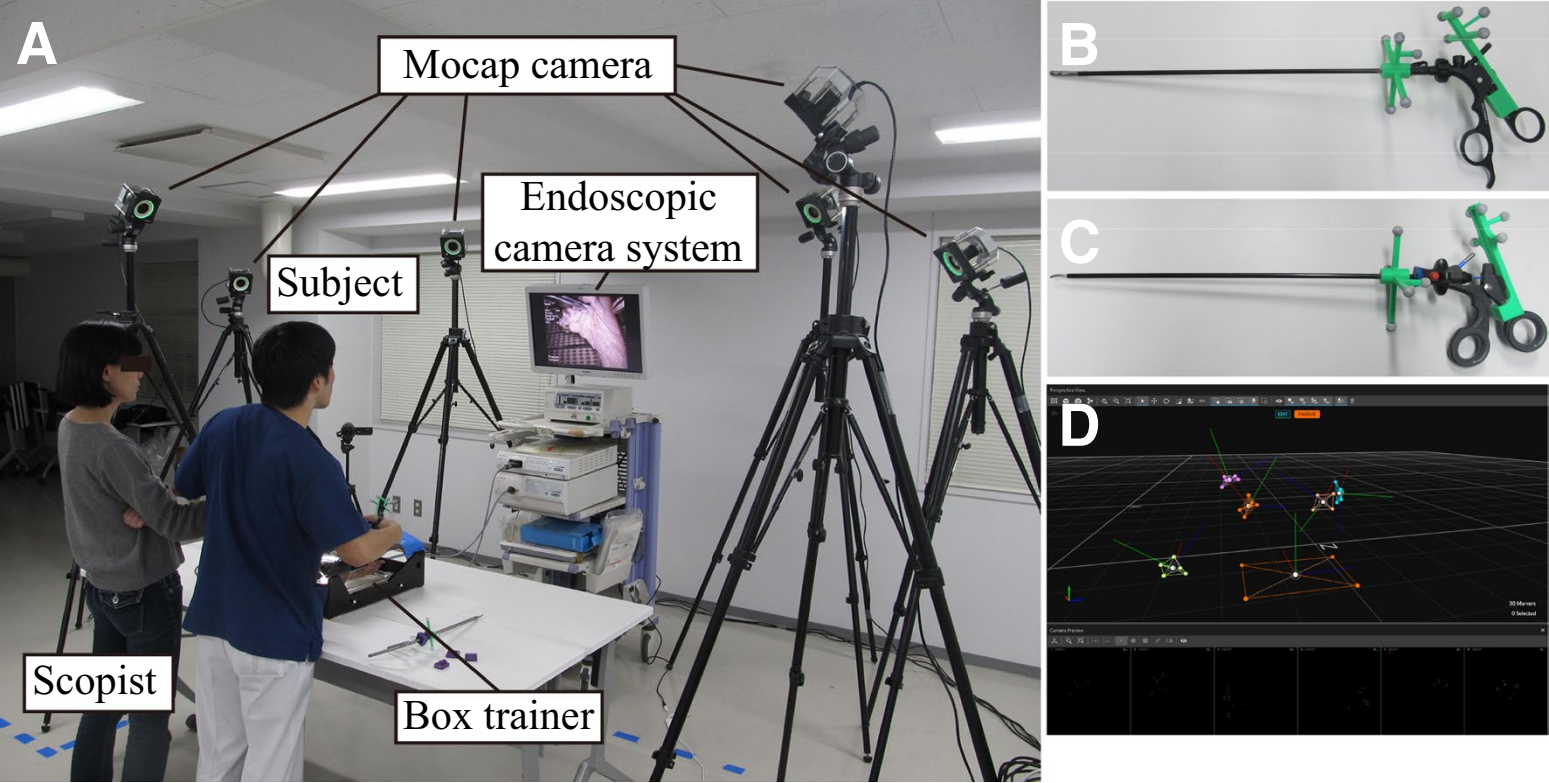
Photographs of the simulation training. **A** The Mocap system, which consisted of six infrared cameras (OptiTrack Prime 41, NaturalPoint Inc., USA), simultaneously tracked the movements of multiple surgical instruments during a series of training steps. **B** grasping forceps, **C** scissors. Infrared reflective marker sets with an individual arrangement pattern were attached to handles of grasping forceps, scissors, a clip applier, and needle holders. **D** computer display showing the movements of surgical devices. The tip movements were calculated based on the positional relationship between the tip and handle

We previously reported the present Mocap system that we newly developed [18]. Briefly, the Mocap system, which consists of six infrared cameras (OptiTrack Prime 41, NaturalPoint Inc., USA), simultaneously tracked the movements of multiple surgical instruments during a series of training steps (Fig. 2A). Infrared reflective marker sets with an individual arrangement pattern were attached to handles of grasping forceps, scissors, a clip applier, and needle holders (Fig. 2B: grasping forceps, Fig. 2C: scissors). Therefore, our system recognized each instrument individually regardless of exchanges of instruments during a procedure. The tip movements were calculated based on the positional relationship between the tip and handle (Fig. 2D: computer display showing the movements of surgical devices). Several markers were also attached to the left and right sides of a box trainer to identify the base position. In our previous study, a questionnaire survey revealed that participants did not feel significant disturbance from the attached marker sets during the manipulation of surgical instruments. The measurement outcomes analyzed in the present study were as follows:

i. Operative time (s): total time to complete a task.
ii. Path length (m): total length of the tip trajectory of an instrument
  $$\sum_{i=1}^{n-1}\sqrt{{\left({x}_{i+1}-{x}_{i}\right)}^{2}+{\left({y}_{i+1}-{y}_{i}\right)}^{2}+{\left({z}_{i+1}-{z}_{i}\right)}^{2}},$$
  where $n$ is the total number of frames, and $x_{i} ,y_{i} ,\,{\text{and}}\,z_{i}$ are tip positions of an instrument in frame $i$. In this study, the trajectory that lies inside the box trainer is the measurement target, excluding that outside of it.
iii. Velocity (cm/s): average velocity of the tip of an instrument.
  $$\frac{1}{n}\sum_{i=1}^{n}\sqrt{{\left(\frac{d{x}_{i}}{dt}\right)}^{2}+{\left(\frac{d{y}_{i}}{dt}\right)}^{2}+{\left(\frac{d{z}_{i}}{dt}\right)}^{2}}$$
iv. Acceleration (cm/s^2^): average acceleration of the tip of an instrument.
  $$\frac{1}{n}\sum_{i=1}^{n}\sqrt{{\left(\frac{{d}^{2}{x}_{i}}{d{t}^{2}}\right)}^{2}+{\left(\frac{{d}^{2}{y}_{i}}{d{t}^{2}}\right)}^{2}+{\left(\frac{{d}^{2}{z}_{i}}{d{t}^{2}}\right)}^{2}}$$
v. Jerk (cm/s^3^): average jerk of the tip of an instrument. Jerk is the changing rate of acceleration, and it represents motion smoothness.
  $$\frac{1}{n}\sum_{i=1}^{n}\sqrt{{\left(\frac{{d}^{3}{x}_{i}}{d{t}^{3}}\right)}^{2}+{\left(\frac{{d}^{3}{y}_{i}}{d{t}^{3}}\right)}^{2}+{\left(\frac{{d}^{3}{z}_{i}}{d{t}^{3}}\right)}^{2}}$$
vi. Frequency of opening and closing (times): total number of iterations of opening and closing the jaws of forceps. “A series of opening and closing the forceps once” is counted as “one iteration”.
vii. Distribution of velocity: the number of frames whose instrument velocity belongs to a certain velocity band as a ratio of the total number of frames $n$. Each velocity band is defined as follows:
  (a) Idle time (%) [0 ≤ to < 0.5 (cm/s)]
  (b) Low-velocity time (%) [0.5 ≤ to < 2.0 (cm/s)]
  (c) Middle-velocity time (%) [2.0 ≤ to < 5.0 (cm/s)]
  (d) High-velocity time (%) [5.0 ≤ to < 12.0 (cm/s)]
  (d) Very high-velocity time (%) [12 ≤ (cm/s)]

viii. Distribution of working area: the path length to a certain area from the target object (Task 1: aorta, Tasks 2 and 3: kidney surface) as a percentage of the total path length. Each working area from the target object is defined as follows:
  Close (%) [0 ≤ to < 2.0 (cm)].
  Near (%) [2.0 ≤ to < 4.0 (cm)].
  Far (%) [4.0 ≤ (cm)].
  In Task 1, at the beginning of training, we intentionally recorded both the starting and ending points (around 17–18-cm distance) by placing both forceps on the aorta for 5 s. In Tasks 2 and 3, the same procedure was performed on the incised line of the kidney parenchyma. Using these data, we defined the concentric cylinder shape of the working area in each case, and calculated the “Distribution of working area” described above.
ix. Average inserting time (s): the inserting time was calculated as the duration between insertion of an applier into a box trainer through a trocar and its removal. The average time was calculated for each case.

In this study, the trajectory of the tip of an instrument ($x_{i} ,y_{i} ,\,{\text{and}}\,z_{i}$) was smoothed by the Savitzky-Golay filter [19], and its derivatives $\left( {\frac{{d^{j} x_{i} }}{{dt^{j} }},\frac{{d^{j} y_{i} }}{{dt^{j} }},{\mkern 1mu} {\text{and}}{\mkern 1mu} \frac{{d^{j} z_{i} }}{{dt^{j} }}\left( {j = 1{\mkern 1mu} {\text{to}}{\mkern 1mu} 3} \right)} \right)$ were also obtained by the filter. The polynomial order of the filter was set to 3, and the number of sampling frames of the filter was set to 31.

## Analyses and statistics

Measurements were compared according to participants’ levels of surgical experience (experts: 50 ≤ laparoscopic surgeries, intermediates: 10–49, novices: 0–9). In the present analyses, we chose a cutoff of 50 cases to define the “expert” category based on a paper demonstrating a shorter operative time on treating over 50 cases [20], an expert opinion [21], and our previous validation study of the present model [1]. The Kruskal–Wallis test was utilized to assess differences among the three groups. If the groups significantly differed, the Mann–Whitney U test was utilized for paired comparison to test the differences between groups. The ESSQ status was also used for comparison. Principal component analysis (PCA) was conducted, a data reduction technique, in order to understand the motion metrics that explained the level of surgical experience in the present tasks. In this study, the metrics showing a significant difference ($p<0.05$) in the Kruskal–Wallis test were used as input data for the analysis. To reduce the effects of outliers, the input data were normalized by a robust Z-score. The normalized data ${z}_{i}$ can be calculated as follows:

$${z}_{i}=\frac{{{X}_{i}-X}_{m}}{NIQR},$$

where, ${X}_{i}$ denotes the original data, ${X}_{m}$ is the median, and $NIQR$ is the normalized interquartile range.

Kruskal–Wallis and Mann–Whitney U tests were performed using JMP 14 (SAS, Japan), and PCA was conducted using R (Ver. 3.6.0).

## Results

Table 1 shows a summary of participants’ backgrounds. Thirty-nine urologists, one junior resident, and five medical students voluntarily participated in the present study. The previous experiences of laparoscopic surgery were as follows: 0–9: *n* = 15, 10–49: *n* = 12, 50–99: *n* = 6, 100–499: *n* = 9, 500 ≤ : *n* = 3. Fifteen participants had the ESSQ qualification. Two surgeons (one expert and one intermediate) were left-handed. However, they were included in the present analysis because they performed actual surgeries with a right-handed style.

**Table 1:** Participants’ characteristics

	*n* = 45
Age, years	Median 33 (range, 23–57)
Sex	Male/Female = 37/8
Background	Urologist, *n* = 39
	Junior resident, *n* = 1
	Medical student, *n* = 5
Experience of laparoscopic surgery	0–9, *n* = 15
	10–49, *n* = 12
	50–99, *n* = 6
	100–499, *n* = 9
	≥ 500, *n* = 3
Endoscopic surgical skill qualification	Yes/No = 15/30
Dominant hand: right/left	43/2

**Fig. 3:**
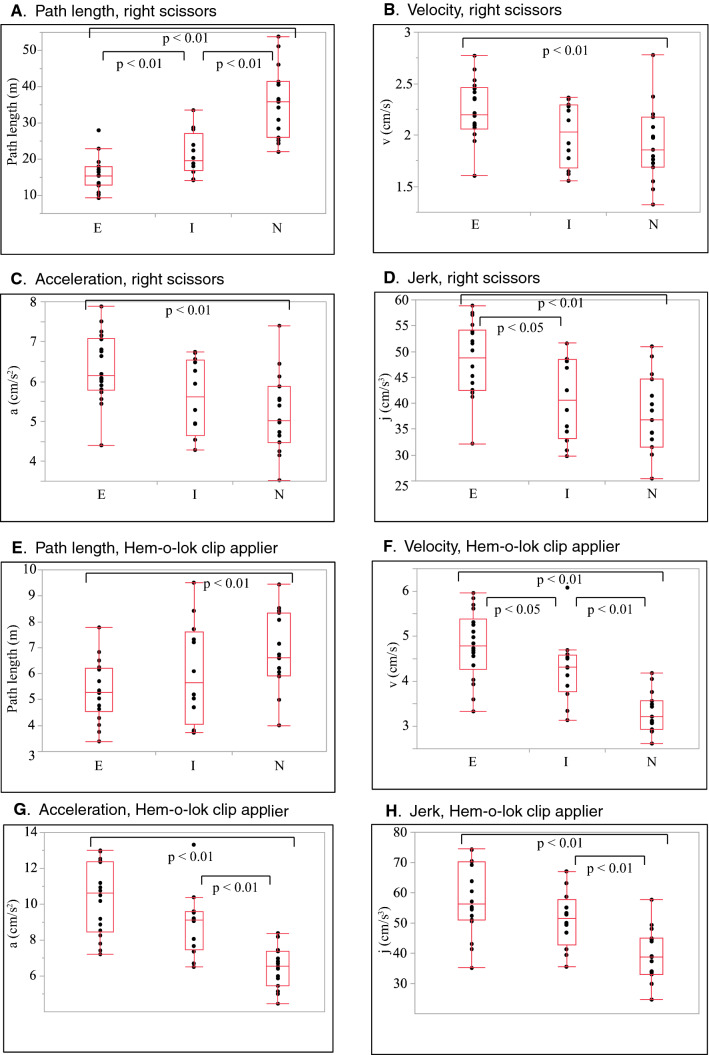
Box plots of path length, velocity, acceleration, and jerk of scissors and the Hem-o-lok clip applier in Task 1 (*E* experts, *I* intermediates, *N* = novices). There were significant differences (*p* < 0.05) in the path length, velocity, acceleration, and jerk among the three groups using both scissors and the Hem-o-lok clip applier, showing the superiority of speed-related parameters and economic movements (shorter path length) of surgical devices managed by the right hand in the more experienced group. Outlier box plots represent the median (center line) and 25th and 75th percentiles (box), and the ends of the whiskers are the outermost data points from their respective quartiles that fall within the distance computed as 1.5 times the interquartile range (IQR). *E* experts, *I* intermediates, *N* novices

**Fig. 4:**
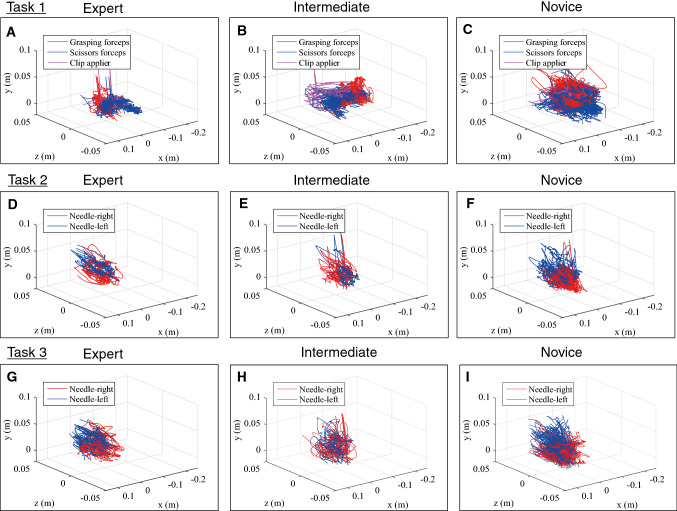
Representative results of trajectory of the instrument tip of an expert, an intermediate, and a novice in the three tasks. The coordinate axis is defined in Fig. 1A and B, and the origin of the coordinate is the starting point of a target object recorded at the beginning of training (see definition of "Distribution of working area" in "Materials and methods"). Overall, a shorter path length was observed in the expert group

Table 2 summarizes the measurement metrics by the present Mocap system divided by the previous surgical experiences. Figure 3 also shows box plots of the path length, velocity, acceleration, and jerk of scissors and the Hem-o-lok clip applier in Task 1. In Task 1, there were significant differences (*p* < 0.05) in the path length, velocity, acceleration, and jerk among the three groups using both scissors and the Hem-o-lok clip applier, showing the superiority of speed-related parameters and economic movements (shorter path length) of surgical devices managed by the right hand of operators in the more experienced group. Regarding the Croce grasping forceps, managed by the left hand, there were significant differences in the path length and frequency of opening and closing, showing economic movements in the more experienced group. In Tasks 2 and 3, there were significant differences in the path length, velocity, and acceleration among the three groups in both hands. Overall, regarding the paired comparisons of the motion metrics showing significant differences on the Kruskal–Wallis test, the difference between experts and novices was large and remained significant, while the difference between novices and intermediates, or that between intermediates and experts was sometimes small and non-significant. Figure 4 shows representative results for the trajectory of the instrument tip of an expert, an intermediate, and a novice in the three tasks. Supplementary Table 1 shows the measurement outcomes divided by the ESSQ qualification status. In Task 1, participants with the ESSQ qualification demonstrated superior speed-related parameters and economic movements with right hand devices, and economic movements with left-hand devices. In Tasks 2 and 3, there were significant differences in the path length and velocity in both hands between participants with and without the ESSQ qualification.

**Table 2:** Summary of measurements by Mocap system

	Novices (experience: 0–9), *n* = 15	Intermediates (experience: 10–49), *n* = 12	Experts (experience: ≥ 50), *n* = 18	*p* Value	N-E	I-E	N-I
*Task 1*							
Operative time (s)	2306.0 (2059.5–2695.2)	1436.4 (970.1–1664.5)	960.0 (796.5–1162.6)	** < 0.0001**	** < 0.0001**	**0.0056**	**0.0010**
* Right hand, scissors*							
Path length (m)	35.8 (27.2–41.0)	19.6 (17.7–25.0)	15.4 (13.1–17.8)	** < 0.0001**	** < 0.0001**	**0.0063**	**0.0007**
Velocity (cm/s)	1.9 (1.7–2.1)	2.0 (1.7–2.3)	2.2 (2.1–2.4)	**0.0184**	**0.0088**	0.0596	0.4208
Acceleration (cm/s^2^)	5.0 (4.6–5.7)	5.6 (4.8–6.5)	6.1 (5.8–7.0)	**0.0067**	**0.0022**	0.0596	0.3413
Jerk (cm/s^3^)	36.8 (32.3–43.1)	40.6 (34.1–48.2)	48.7 (42.6–53.8)	**0.0018**	**0.0006**	**0.0262**	0.3413
Frequency of opening and closing (number of iterations) (times)	364.0 (323.0–443.5)	236.0 (159.8–278.0)	213.0 (148.3–238.8)	**0.0028**	**0.0019**	0.1892	**0.0179**
Distribution of velocity							
Idle time (%) [0 ≤ to < 0.5 (cm/s)]	13.0 (9.8–14.8)	11.9 (9.5–14.4)	7.6 (6.7–9.7)	**0.0038**	**0.0014**	**0.0325**	0.4495
Low-velocity time (%) [0.5 ≤ to < 2.0 (cm/s)]	51.7 (44.5–56.9)	49.4 (43.7–55.8)	45.4 (43.1–50.7)	0.2204			
Middle-velocity time (%) [2.0 ≤ to < 5.0 (cm/s)]	32.0 (31.2–36.7)	35.2 (29.9–39.6)	39.1 (34.8–42.2)	**0.0316**	**0.0108**	0.1031	0.5747
High-velocity time (%) [5.0 ≤ to < 12.0 (cm/s)]	3.3 (1.7–6.0)	4.7 (3.2–6.9)	5.5 (4.4–7.9)	0.0635			
Very high-velocity time (%) [12.0 ≤ (cm/s)]	0.0 (0.0–0.1)	0.0 (0.0–0.1)	0.2 (0.1–0.2)	0.1114			
Distribution of working area from aorta							
Close zone (%) [0 ≤ to < 2.0 (cm)]	67.2 (61.6–72.9)	61.0 (57.5–73.6)	80.0 (62.1–84.9)	0.1747			
Near zone (%) [2.0 ≤ to < 4.0 (cm)]	31.5 (24.5–37.2)	37.3 (27.0–40.2)	20.1 (13.6–34.9)	0.1596			
Far zone (%) [4.0 ≤ (cm)]	1.8 (1.1–3.5)	2.3 (1.6–3.3)	1.9 (1.1–3.0)	0.4361			
* Right hand, Hem-o-lok*							
Path length (m)	6.6 (6.0–8.2)	5.6 (4.5–7.4)	5.3 (4.7–6.2)	**0.0350**	**0.0051**	0.5677	0.2723
Velocity (cm/s)	3.2 (3.0–3.5)	4.3 (3.9–4.5)	4.8 (4.4–5.3)	** < 0.0001**	** < 0.0001**	**0.0401**	**0.0007**
Acceleration (cm/s^2^)	6.5 (5.7–7.2)	9.1 (7.6–9.6)	10.6 (8.6–12.1)	** < 0.0001**	** < 0.0001**	0.0720	**0.0010**
Jerk (cm/s^3^)	38.8 (33.4–44.7)	51.4 (45.5–56.0)	56.2 (51.4–70.1)	** < 0.0001**	**0.0001**	0.0720	**0.0032**
Average inserting time (s)	17.7 (15.0–18.8)	12.2 (11.0–13.1)	9.4 (8.7–10.6)	** < 0.0001**	** < 0.0001**	**0.0049**	**0.0005**
Distribution of velocity							
Idle time (%) [0 ≤ to < 0.5 (cm/s)]	19.4 (16.9–23.5)	14.7 (12.2–18.5)	11.1 (8.8–17.3)	**0.0077**	**0.0025**	0.4091	**0.0481**
Low-velocity time (%) [0.5 ≤ to < 2.0 (cm/s)]	45.5 (43.4–50.1)	45.2 (42.4–46.6)	44.8 (41.1–47.7)	0.4796			
Middle-velocity time (%) [2.0 ≤ to < 5.0 (cm/s)]	15.7 (14.3–19.6)	18.6 (17.7–21.3)	20.9 (17.1–22.9)	**0.0394**	**0.0179**	0.6264	0.0673
High-velocity time (%) [5.0 ≤ to < 12.0 (cm/s)]	8.9 (7.9–10.1)	12.3 (8.5–14.0)	11.1 (8.3–13.2)	0.1973			
Very high-velocity time (%) [12.0 ≤ (cm/s)]	7.1 (6.2–7.9)	9.9 (8.6–10.4)	11.1 (9.9–12.5)	** < 0.0001**	** < 0.0001**	0.0720	**0.0023**
Distribution of working area from aorta							
Close zone (%) [0 ≤ to < 2.0 (cm)]	15.2 (11.9–18.6)	10.3 (7.6–16.9)	15.9 (11.2–19.0)	0.1991			
Near zone (%) [2.0 ≤ to < 4.0 (cm)]	29.0 (22.5–31.7)	27.3 (23.1–30.5)	18.3 (16.8–25.6)	**0.0123**	**0.0051**	0.0720	0.2941
Far zone (%) [4.0 ≤ (cm)]	56.8 (53.8–59.7)	63.2 (58.9–66.5)	65.1 (61.5–66.1)	**0.0039**	**0.0014**	0.7190	**0.0205**
*Left hand, Croce grasping forceps*							
Path length (m)	21.3 (17.8–25.7)	12.8 (11.3–13.6)	8.1 (6.4–10.8)	** < 0.0001**	** < 0.0001**	**0.0235**	** < 0.0001**
Velocity (cm/s)	1.0 (0.8–1.2)	0.9 (0.7–1.1)	0.9 (0.8–1.1)	0.6755			
Acceleration (cm/s^2^)	2.5 (2.0–3.0)	2.2 (1.7–2.9)	2.4 (2.1–2.9)	0.6813			
Jerk (cm/s^3^)	17.9 (14.3–21.3)	14.9 (12.1–20.6)	17.1 (15.1–21.4)	0.6199			
Frequency of opening and closing (number of iterations) (times)	93.0 (82.5–125.0)	53.0 (46.5–61.5)	36.5 (27.3–52.3)	**0.0004**	**0.0004**	0.0654	**0.0078**
Distribution of velocity							
Idle time (%) [0 ≤ to < 0.5 (cm/s)]	55.9 (49.6–57.2)	63.5 (53.9–67.4)	57.6 (46.6–63.3)	0.2046			
Low-velocity time (%) [0.5 ≤ to < 2.0 (cm/s)]	31.4 (29.9–33.2)	27.5 (23.3–29.0)	30.9 (27.7–35.9)	**0.0264**	1.0000	**0.0235**	**0.0137**
Middle-velocity time (%) [2.0 ≤ to < 5.0 (cm/s)]	10.9 (8.8–15.5)	9.8 (6.8–14.3)	10.2 (7.5–12.1)	0.5128			
High-velocity time (%) [5.0 ≤ to < 12.0 (cm/s)]	2.1 (1.1–3.4)	2.0 (1.0–3.8)	2.1 (1.7–2.6)	0.9988			
Very high-velocity time (%) [12.0 ≤ (cm/s)]	0.1 (0.0–0.1)	0.1 (0.0–0.2)	0.1 (0.0–0.2)	0.8983			
Distribution of working area from aorta							
Close zone (%) [0 ≤ to < 2.0 (cm)]	36.0 (30.3–39.9)	37.6 (30.8–42.3)	29.6 (17.7–41.0)	0.2635			
Near zone (%) [2.0 ≤ to < 4.0 (cm)]	48.2 (44.4–55.8)	42.8 (39.8–49.5)	49.0 (37.0–52.2)	0.4572			
Far zone (%) [4.0 ≤ (cm)]	12.2 (6.2–29.3)	19.3 (11.2–23.0)	20.4 (6.5–35.8)	0.5265			
*Task 2*							
Operative time (s)	406.9 (279.6–579.3)	289.2 (178.8–345.1)	167.7 (142.7–185.1)	** < 0.0001**	** < 0.0001**	**0.0235**	**0.0233**
*Right hand, needle holder*							
Path length (m)	6.2 (4.2–9.3)	4.2 (3.0–5.4)	3.4 (2.6–3.9)	**0.0021**	**0.0006**	0.1966	**0.0481**
Velocity (cm/s)	1.5 (1.4–1.7)	1.7 (1.6–1.8)	1.9 (1.8–2.1)	**0.0028**	**0.0022**	**0.0210**	0.1500
Acceleration (cm/s^2^)	3.8 (3.6–4.3)	4.3 (4.0–4.8)	4.9 (4.4–5.3)	**0.0090**	**0.0051**	**0.0443**	0.2515
Jerk (cm/s^3^)	30.4 (27.6–32.7)	32.7 (29.2–35.2)	35.5 (31.1–41.0)	0.0572			
Distribution of velocity							
Idle time (%) [0 ≤ to < 0.5 (cm/s)]	20.8 (16.8–22.8)	18.5 (16.1–22.6)	18.2 (13.7–21.8)	0.4629			
Low-velocity time (%) [0.5 ≤ to < 2.0 (cm/s)]	55.9 (51.3–58.1)	52.1 (49.1–54.3)	49.0 (47.5–50.0)	**0.0004**	**0.0001**	0.0541	0.0603
Middle-velocity time (%) [2.0 ≤ to < 5.0 (cm/s)]	21.7 (19.0–23.8)	25.8 (23.5–27.8)	27.3 (24.1–30.7)	**0.0034**	**0.0016**	0.1966	**0.0338**
High-velocity time (%) [5.0 ≤ to < 12.0 (cm/s)]	1.9 (1.1–3.2)	3.9 (2.6–4.7)	5.0 (4.2–6.6)	**0.0011**	**0.0006**	**0.0443**	0.0603
Very high-velocity time (%) [12.0 ≤ (cm/s)]	0.3 (0.1–0.4)	0.0 (0.0–0.2)	0.2 (0.0–0.7)	0.1437			
Distribution of working area from kidney							
Close zone (%) [0 ≤ to < 2.0 (cm)]	34.3 (25.5–51.6)	37.4 (29.9–42.8)	32.7 (23.1–46.0)	0.8249			
Near zone (%) [2.0 ≤ to < 4.0 (cm)]	49.4 (41.5–54.7)	50.5 (43.6–55.2)	43.2 (39.0–53.2)	0.5438			
Far zone (%) [4.0 ≤ (cm)]	15.8 (6.3–19.2)	12.2 (7.9–14.2)	23.3 (9.8–26.6)	0.1348			
* Left hand, needle holder*							
Path length (m)	5.5 (4.0–8.3)	4.0 (3.1–5.6)	2.8 (2.2–3.4)	**0.0040**	**0.0014**	0.0945	0.1243
Velocity (cm/s)	1.4 (1.4–1.5)	1.6 (1.4–1.8)	1.7 (1.6–2.0)	**0.0365**	**0.0088**	0.3627	0.2319
Acceleration (cm/s^2^)	3.7 (3.6–4.0)	4.2 (3.7–5.0)	4.6 (4.1–5.1)	**0.0479**	**0.0197**	0.3855	0.1243
Jerk (cm/s^3^)	28.7 (25.8–30.7)	34.3 (27.0–38.7)	33.3 (29.1–38.2)	0.1333			
Distribution of velocity							
Idle time (%) [0 ≤ to < 0.5 (cm/s)]	30.1 (25.1–32.7)	27.7 (17.8–35.4)	23.3 (20.3–30.9)	0.2401			
Low-velocity time (%) [0.5 ≤ to < 2.0 (cm/s)]	47.0 (41.7–49.6)	43.6 (40.2–48.7)	45.6 (40.8–50.3)	0.7197			
Middle-velocity time (%) [2.0 ≤ to < 5.0 (cm/s)]	21.1 (18.5–23.9)	24.4 (18.4–27.6)	23.5 (21.7–27.5)	0.0837			
High-velocity time (%) [5.0 ≤ to < 12.0 (cm/s)]	3.1 (2.1–3.6)	3.2 (2.0–6.6)	3.7 (2.8–5.3)	0.3171			
Very high-velocity time (%) [12.0 ≤ (cm/s)]	0.1 (0.0–0.2)	0.0 (0.0–0.4)	0.2 (0.0–0.6)	0.5000			
Distribution of working area from kidney							
Close zone (%) [0 ≤ to < 2.0 (cm)]	36.9 (27.7–40.5)	33.1 (25.2–40.7)	25.5 (14.8–36.7)	0.1374			
Near zone (%) [2.0 ≤ to < 4.0 (cm)]	48.5 (45.1–53.4)	48.5 (43.6–57.1)	50.2 (44.5–56.2)	0.9283			
Far zone (%) [4.0 ≤ (cm)]	17.5 (10.6–21.8)	14.4 (7.3–21.3)	24.7 (9.3–34.5)	0.2109			
*Task 3*							
Operative time (s)	603.9 (387.2–836.0)	357.4 (296.7–524.7)	270.5 (253.8–297.3)	** < 0.0001**	** < 0.0001**	**0.0262**	**0.0157**
*Right hand, needle holder*							
Path length (m)	10.5 (7.9–15.7)	7.4 (5.8–9.2)	5.6 (5.3–6.2)	**0.0014**	**0.0011**	**0.0361**	0.0539
Velocity (cm/s)	1.9 (1.6–2.0)	2.1 (1.8–2.3)	2.2 (2.1–2.5)	**0.0017**	**0.0006**	0.1031	0.0749
Acceleration (cm/s^2^)	4.6 (3.9–5.2)	5.3 (4.6–5.7)	5.5 (5.2–6.8)	**0.0054**	**0.0018**	0.1442	0.1021
Jerk (cm/s^3^)	32.4 (29.2–35.9)	39.0 (33.1–41.9)	40.1 (35.4–48.5)	**0.0078**	**0.0020**	0.2117	0.1243
Distribution of velocity							
Idle time (%) [0 ≤ to < 0.5 (cm/s)]	18.4 (15.7–21.1)	18.4 (13.9–20.8)	15.0 (13.2–16.1)	0.0758			
Low-velocity time (%) [0.5 ≤ to < 2.0 (cm/s)]	47.3 (46.2–51.7)	45.9 (42.3–48.3)	43.1 (41.4–45.9)	**0.0043**	**0.0012**	0.2117	0.0749
Middle-velocity time (%) [2.0 ≤ to < 5.0 (cm/s)]	28.8 (24.3–31.7)	29.7 (25.9–31.5)	32.8 (30.7–35.3)	**0.0148**	**0.0071**	0.0596	0.3413
High-velocity time (%) [5.0 ≤ to < 12.0 (cm/s)]	4.3 (2.9–5.9)	6.7 (5.4–8.3)	8.2 (6.2–9.1)	**0.0009**	**0.0002**	0.2619	**0.0299**
Very high-velocity time (%) [12.0 < (cm/s)]	0.2 (0.1–0.3)	0.2 (0.1–0.4)	0.4 (0.3–0.9)	**0.0195**	**0.0162**	**0.0235**	0.8262
Distribution of working area from kidney							
Close zone (%) [0 ≤ to < 2.0 (cm)]	30.3 (18.1–42.9)	26.4 (18.7–34.0)	28.7 (18.7–32.9)	0.8905			
Near zone (%) [2.0 ≤ to < 4.0 (cm)]	45.6 (41.9–49.2)	52.7 (50.6–54.7)	48.9 (45.7–53.5)	0.0605			
Far zone (%) [4.0 ≤ (cm)]	21.1 (13.8–32.8)	22.6 (11.0–26.7)	19.1 (16.7–27.9)	0.8758			
* Left hand, needle holder*							
Path length (m)	8.8 (6.9–14.9)	6.4 (5.9–8.1)	5.0 (4.4–5.6)	**0.0025**	**0.0018**	**0.0401**	0.0749
Velocity (cm/s)	1.7 (1.6–1.7)	1.8 (1.7–1.9)	2.0 (1.8–2.4)	**0.0218**	**0.0097**	0.1329	0.1796
Acceleration (cm/s^2^)	4.2 (3.9–4.6)	4.4 (4.1–5.0)	5.1 (4.3–6.2)	**0.0397**	**0.0179**	0.1689	0.2134
Jerk (cm/s^3^)	29.2 (27.4–33.2)	31.5 (27.9–36.2)	34.4 (30.1–43.2)	0.0745			
Disribution of velocity							
Idle time (%) [0 ≤ to < 0.5 (cm/s)]	24.2 (20.9–25.9)	22.6 (19.3–24.5)	21.1 (16.0–23.8)	0.1630			
Low-velocity time (%) [0.5 ≤ to < 2.0 (cm/s)]	46.6 (43.1–47.8)	44.7 (39.7–48.7)	44.7 (40.5–46.1)	0.4085			
Middle-velocity time (%) [2.0 ≤ to < 5.0 (cm/s)]	24.9 (23.1–28.3)	28.3 (25.4–30.0)	29.7 (26.5–32.0)	0.0616			
High-velocity time (%) [5.0 ≤ to < 12.0 (cm/s)]	4.1 (3.1–5.3)	4.5 (3.7–7.0)	4.9 (4.2–8.9)	0.1396			
Very high-velocity time (%) [12.0 ≤ (cm/s)]	0.1 (0.0–0.3)	0.1 (0.0–0.2)	0.4 (0.0–0.7)	0.1051			
Distribution of working area from kidney							
Close zone (%) [0 ≤ to < 2.0 (cm)]	26.2 (16.7–43.3)	26.1 (20.9–30.8)	19.4 (13.8–32.0)	0.3678			
Near zone (%) [2.0 ≤ to < 4.0 (cm)]	50.0 (41.5–52.7)	49.8 (46.7–56.5)	51.7 (46.2–59.3)	0.2685			
Far zone (%) [4.0 ≤ (cm)]	17.9 (11.7–34.9)	16.7 (13.7–27.3)	21.5 (16.0–35.4)	0.5378			

Bold values are statistically significant (*p* < 0.05)

Median [interquartile range]

*E* experts, *I* intermediates, *N* novices

**Fig. 5:**
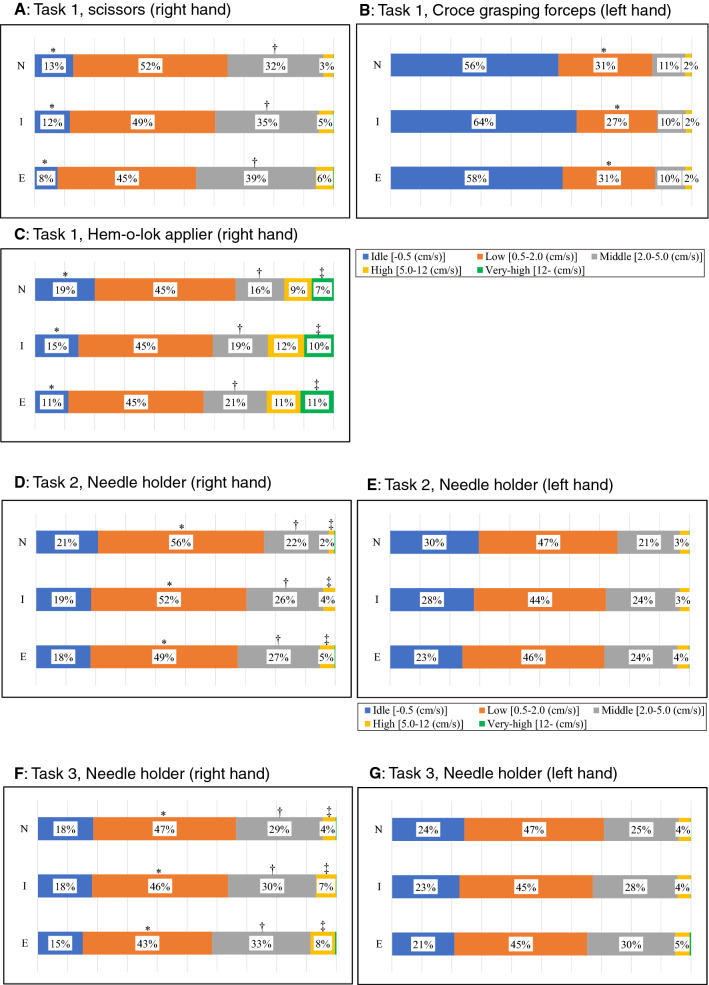
Bar charts showing the distribution of the median velocity for each instrument among the three groups (*E* experts, *I* intermediates, *N* = novices). Overall, there was a trend toward a shorter idle state and longer state of the higher velocity range in the expert group for all instruments except the Croce grasping forceps used in Task 1. Proportion less than 1% of the very high-velocity range was not described in the figure. *, †, and ‡ indicates statistically significant (*p* < 0.05) among the 3 groups.  *E* experts, *I* intermediates, *N* = novices

**Fig. 6:**
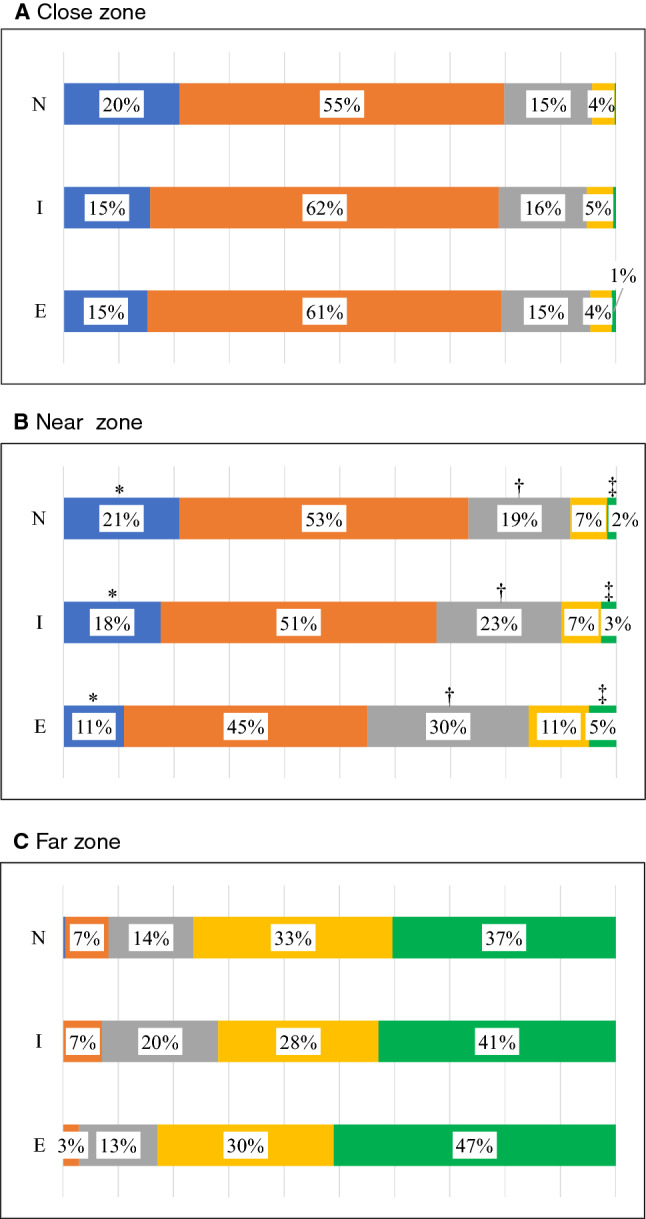
Sub-analysis of the velocity range of the Hem-o-lok clip applier according to the working area (*E* experts, *I* intermediates, *N* = novices). A shorter idle state and quicker movements were significantly observed in the “near zone. Proportion less than 1% of the very high-velocity range was not described in the figure. *, †, and ‡ indicates statistically significant (*p* < 0.05) among the 3 groups.  *E* experts, *I* intermediates, *N* = novices

Using data in Table 2, we created bar charts showing the distribution of the median velocity of each instrument among the three groups (Fig. 5). Overall, there was a trend toward a shorter idle state and longer state of the quicker velocity range in the expert group for all instruments excluding the Croce grasping forceps used in Task 1, and significant differences in the ratio of the velocity range were frequently observed in instruments managed by the right hand. Regarding the Hem-o-lok clip applier, sub-analysis of the ratio of the velocity range according to the working area showed significantly shorter idle states and quicker movements in the “near zone (2.0 ≤ – < 4.0 cm from aorta)” in the expert group (Fig. 6). Regarding analysis of the distribution of the working area, the trajectory of the Hem-o-lok clip applier was longer in the “near zone (2.0 ≤ – < 4.0 cm from aorta)” in novice and intermediate groups (novices: median 29.0%, intermediates: median 27.3%, experts: median 18.3%, *p* = 0.0123), which suggested the hovering of the instrument before determining the closure site on the vascular pedicle.

We further compared the metrics between the experts with 50–99 surgical cases and those with more than 100 cases (Table 3). In Task 1, right hand scissors moved more frequently in the “close zone (0 ≤ – < 2.0 cm from aorta)” in experts with more than 100 cases (experts 100 ≤ cases: 83.9%, experts of 50–99: 60.5%, p = 0.0145). In Task 3, experts with 100 ≤ cases showed a shorter operative time and shorter path length for the right hand needle holder. There was no significant difference in any parameters in Task 2.

**Table 3:** Comparative study of Mocap metrics between the experts with 50–99 surgical cases and those with more than 100 cases

	Experts (experience: ≥ 100), *n* = 12	Experts (experience: 50–99), *n* = 16	*p*-Value
*Task 1*			
Operative time (s)	921.1 (781.0–1037.8)	1015.6 (798.3–1218.6)	0.5636
*Right hand, scissors*			
Path length (m)	15.4 (13.5–17.0)	14.5 (12.9–18.3)	0.9646
Velocity (cm/s)	2.3 (2.1–2.4)	2.1 (2.1–2.4)	0.6251
Acceleration (cm/s^2^)	6.5 (6.0–7.0)	6.0 (5.8–6.8)	0.5636
Jerk (cm/s^3^)	50.9 (43.3–54.8)	45.6 (42.5–52.5)	0.6893
Frequency of opening and closing (number of iteration) (times)	216.0 (155.3–236.0)	200.0 (140.5–241.8)	0.7556
Distribution of velocity			
Idle time (%) [0 ≤ to < 0.5 (cm/s)]	6.9 (6.7–8.2)	8.6 (7.4–10.6)	0.4501
Low-velocity time (%) [0.5 ≤ to < 2.0 (cm/s)]	44.7 (42.8–48.5)	49.4 (43.2–51.0)	0.4501
Middle-velocity time (%) [2.0 ≤ to < 5.0 (cm/s)]	40.4 (38.0–42.3)	36.8 (34.6–40.6)	0.4501
High-velocity time (%) [5.0 ≤ to < 12.0 (cm/s)]	6.6 (4.7–7.9)	5.0 (4.4–6.4)	0.5052
Very high-velocity time (%) [12.0 ≤ (cm/s)]	0.2 (0.1–0.2)	0.1 (0.0–0.3)	0.8939
Distribution of working area from aorta			
Close zone (%) [0 ≤ to < 2.0 (cm)]	83.9 (80.2–86.0)	60.5 (45.5–79.8)	**0.0145**
Near zone (%) [2.0 ≤ to < 4.0 (cm)]	16.0 (12.6–19.7)	34.5 (20.1–53.5)	**0.0295**
Far zone (%) [4.0 ≤ (cm)]	1.9 (0.9–2.9)	2.2 (1.6–3.2)	0.6893
*Task 3*			
Operative time (s)	258.7 (225.8–272.6)	303.7 (279.4–371.7)	**0.0456**
*Right hand, needle holder*			
Path length (m)	5.4 (4.9–5.9)	6.1 (5.6–9.5)	**0.0456**
Velocity (cm/s)	2.2 (2.2–2.4)	2.2 (2.1–2.6)	0.6251
Acceleration (cm/s^2^)	5.5 (5.2–6.0)	6.0 (5.3–6.9)	0.6893
Jerk (cm/s^3^)	39.3 (35.4–40.7)	44.8 (35.5–50.5)	0.3986
Distribution of velocity			
Idle time (%) [0 ≤ to < 0.5 (cm/s)]	14.5 (11.1–17.7)	15.3 (13.4–16.0)	0.7558
Low-velocity time (%) [0.5 ≤ to < 2.0 (cm/s)]	42.2 (40.0–44.6)	45.5 (42.6–47.3)	0.2303
Middle-velocity time (%) [2.0 ≤ to < 5.0 (cm/s)]	33.3 (30.7–36.5)	32.0 (30.2–33.6)	0.5052
High-velocity time (%) [5.0 ≤ to < 12.0 (cm/s)]	8.5 (6.5–9.5)	7.4 (5.9–8.9)	0.4501
Very high-velocity time (%) [12.0 ≤ (cm/s)]	0.4 (0.3–0.6)	0.7 (0.3–0.9)	0.6251
Distribution of working area from kidney			
Close zone (%) [0 ≤ to < 2.0 (cm)]	21.6 (17.8–31.5)	31.7 (25.7–35.2)	0.1684
Near zone (%) [2.0 ≤ to < 4.0 (cm)]	48.9 (47.0–53.0)	47.9 (44.3–53.1)	0.7558
Far zone (%) [4.0 ≤ (cm)]	21.7 (19.1–32.1)	17.9 (16.1–19.9)	0.2303

Bold values are statistically significant (*p* < 0.05)

**Fig. 7:**
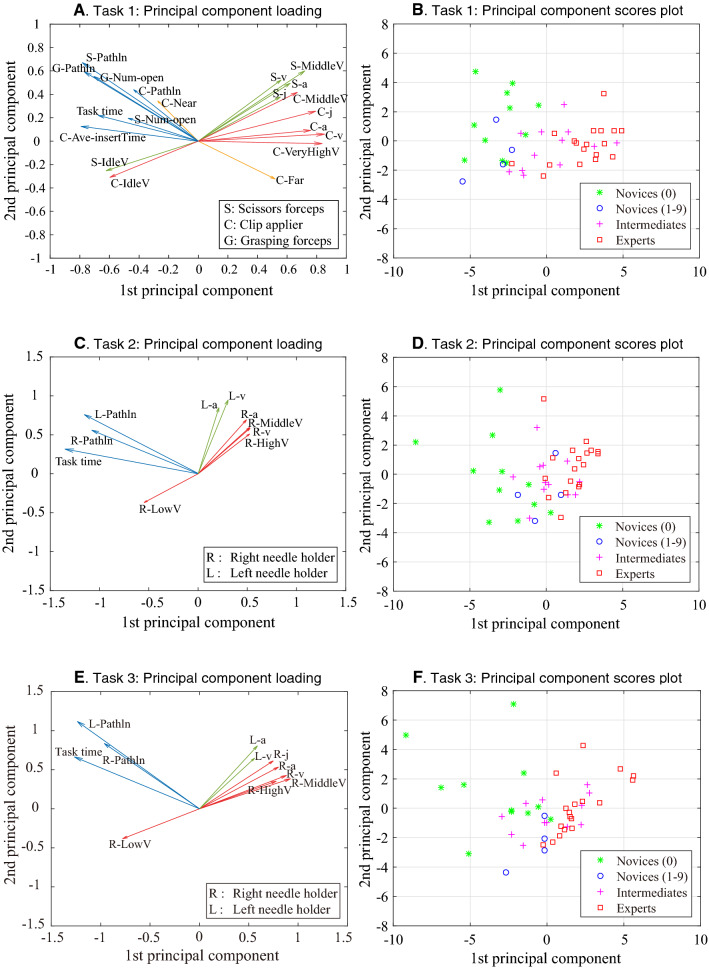
Principal component analysis regarding the level of surgical experience. **A** Loading plots of 1st and 2nd principal components of Task 1. S-Pathln = path length of scissors, G-Pathln = path length of grasping forceps, G-Num-open = frequency of opening and closing grasping forceps, C-Pathln = path length of Hem-o-lok clip applier, C-Ave-insertTime = average inserting time of Hem-o-lok clip applier, C-Near = distribution of working area, near zone of Hem-o-lok clip applier, S-Num-open  = frequency of opening and closing of scissors, S-IdleV = distribution of velocity, idle time of scissors, C-IdleV = distribution of velocity, idle time of Hem-o-lok clip applier, S-MiddleV = distribution of velocity, middle-velocity time of scissors, S-v = velocity of scissors, S-a = acceleration of scissors, S-j = jerk of scissors, C-MiddleV = distribution of velocity, middle-velocity time of Hem-o-lok clip applier, C-j = jerk of Hem-o-lok clip applier, C-a = acceleration of Hem-o-lok-clip applier, C-v = velocity of Hem-o-lok clip applier, C-VeryHighV = distribution of velocity, very high-velocity of Hem-o-lok clip applier, C-Far  = distribution of working area from Aorta, far zone of Hem-o-lok applier. **B** Score plots of 1st and 2nd principal components of Task 1. **C** Loading plots of 1st and 2nd principal components of Task 2. L-Pathln = path length of left needle holder, R-Pathln = path length of right needle holder, R-LowV = distribution of velocity, low-velocity time of right needle holder, L-a = acceleration of left needle holder, L-v = velocity of left needle holder, R-a = acceleration of right needle holder, R-MiddleV = distribution of velocity, middle-velocity time of right needle holder, R-v = velocity of right needle holder, R-HighV = distribution of velocity, high-velocity time of right needle holder. **D** Score plots of 1st and 2nd principal components of Task 1. **E** Loading plots of 1st and 2nd principal components of Task 3. L-Pathln = path length of left needle holder, R-Pathln = path length of right needle holder, R-LowV = distribution of velocity, low-velocity time of right needle holder, L-a = acceleration of left needle holder, L-v = velocity of left needle holder, R-j = jerk of right needle holder, R-a = acceleration of right needle holder, R-v = velocity of right needle holder, R-MiddleV = distribution of velocity, middle-velocity time of right needle holder, R-HighV = distribution of velocity, high-velocity time of right needle holder. **F** Score plots of 1st and 2nd principal components of Task 3

Figure 7 shows the results of PCA regarding the level of surgical experience. Figure 7A, C, and E shows loading plots of 1st and 2nd principal components of each task, and Fig. 7B, D, and F shows score plots of 1st and 2nd principal components, respectively. In Task 1, we did not include the “Low-velocity time of Croce grasping forceps” for the PCA because it showed a V shape among the three groups, and not a constant tendency according to surgical experience, and in Task 3, we did not include the “Very high-velocity time” because the calculated values were extremely low (0–0.7%). As shown in Fig. 7A, C, and E, the principal loading vectors were roughly distributed in two directions. The right-directed vectors were associated with speed-related metrics (e.g., velocity and acceleration, shown by red and green arrows, respectively), and the left-directed vectors were associated with efficiency-related metrics (e.g., pathlength, task time, and frequency of opening and closing, shown by blue arrows). In Task 1, both categories contributed to the axes of 1st and 2nd principal components and Hem-o-lok clip applier-related metrics strongly contributed to the axis of the 1st principal component (Fig. 7A). In Task 2, efficiency-related metrics (path length and task time) strongly contributed to the axis of the 1st principal component (Fig. 7C), while both categories did in Task 3 (Fig. 7E). In Task 1, 68% of the total variance was explained by the 1st and 2nd principal components, and in Tasks 2 and 3, 81 and 82% of the total variance was explained by the 1st and 2nd principal component, respectively. Figure 7B, D, and F shows principal component score plots for each task. Overall, experts’ scores were distributed in the right zone, intermediates’ scores in the middle, and novices’ scores in the left zone in all three Tasks. In addition, the plots of novices without any surgical experience [Novice (0)] were mainly distributed in the leftmost area, which meant the lowest skill level zone. Several participants were distributed in a different category-zone from that expected according to the level of surgical experience.

## Discussion

To our knowledge, this is the first study of motion tracking of multiple surgical instruments simultaneously in a series of training drills using an animal organ model. As described in Materials and Methods, because our system can recognize each instrument individually regardless of exchanges of instruments during a procedure, it facilitated the present Mocap analyses of relatively complicated surgical tasks.

Regarding Task 1 (tissue dissection around aorta), it was developed to help young trainees learn laparoscopic dissection skills around a vessel and Hem-o-lok clip application on a vessel. Trainees were required to remove the tissue around the aorta, dividing encountered mesenteric vessels after applying a Hem-o-lok, which means that trainees need to frequently exchange the instruments according to the situation. As shown in Table 2 and Fig. 3, superior speed-related parameters (velocity, acceleration, and jerk) and economic movements (shorter path length) involving scissors managed by the right hand, and economic movements (shorter pathlength and lower frequency of opening and closing) involving Croce grasping forceps managed by the left hand were observed. Furthermore, although “applying a Hem-o-lok” was a quick procedure, there were significant differences in the path length, velocity, acceleration, jerk, and average inserting time among the three groups. When applying a Hem-o-lok, surgeons were required to feed an applier toward the objective vessel without bumping or injuring intervening obstacles, ideally along the shortest route, close the Hem-o-lok, and remove the applier in reverse along the same route. After that, surgeons needed to bring the scissors back to the working area in the same manner, which partially influenced the total path length of scissors. Our observations strongly suggest that getting surgical devices in/out smoothly and correctly using a limited and two-dimensional monitor, namely safe and efficient exchange of surgical instruments with limited visual information, requires highly trained visual spatial skills, and it well-reflected the level of surgical experience in laparoscopy. We consider that a training task designed to learn visual spatial skills to exchange surgical instruments safely should be included in a laparoscopic training curriculum. Regarding the suturing/knot tying tasks (Tasks 2 and 3), our observations were in line with previous findings that a shorter task time, shorter path length, and faster velocity were observed in an expert group.

Regarding the distribution of the working area, Buckley et al. previously reported “zone” metrics, defined as the percentage of time spent with the instruments within pre-defined areas. In their study, ten medical students, ten surgical residents, and five experts performed a laparoscopic suturing task using ProMIS III® Simulator, and there was a significant difference in the average “in-zone (0–6 cm) score” among the three groups. The average right/left in-zone scores were 88/83% for experts, 72/69% for surgical residents, and 49/50% for medical students [11]. In the present study, we directly calculated the ratio of the path length of a certain area from the target object (Task 1: aorta, Tasks 2 and 3: kidney surface) to the total path length. Regarding suturing/knot tying tasks (Tasks 2 and 3), we did not observe a significant difference in the distribution of the working area among the three groups (Table 2). Difference in the definition of parameters [time-basis (Buckley et al.) vs. path length-basis (ours)], that of the training task [one point suture/knot tying (Buckley et al.) vs. two points (Task 3 in ours)], and that of materials [non-biological material (Buckley et al.) vs. swine kidney (ours)] might have influenced our observations. Rather, in Task 1, we observed that the trajectory of the Hem-o-lok applier was longer in the “near zone (2.0 ≤ to < 4.0 cm from aorta)” in novice and intermediate groups, which suggested the hovering of the instrument before determining the closure site on the vascular pedicle. Furthermore, we observed that experts with more than 100 cases handled scissors more dexterously in the “close zone (0  ≤ to < 2.0 cm from aorta)”, which suggested short, deft movements around the objectives. On considering the results together with the study of Buckley et al., “zone-metrics” may be a promising parameter associated with the level of surgical expertise, and a future study is necessary to confirm its validity.

The idle time means the time period when instrument movement/interaction is minimal. Previous studies showed significant differences in idle times between a novice surgeon and an experienced surgeon regarding laparoscopic suturing, a more complex procedure, and an open surgery suturing task [22–24]. These results can be interpreted as a novice surgeon needs more time for motor planning and decision-making than a more experienced surgeon. As shown in Fig. 5, our results also showed a trend toward a shorter idle state and longer state of the quicker velocity range in the expert group for all instruments except the Croce grasping forceps, and this was more apparent for instruments managed in the right hand. Finally, in order to simplify data and identify the most relevant motion metrics that differentiated levels of surgical experience, we performed PCA analysis of the measured data. Regarding the PCA scores plot, experts’ scores were generally distributed in the right zone, intermediates’ scores in the middle, and novices’ scores in the left zone in all three Tasks. Several participants were in a different category-zone from that expected according to the level of surgical experience, which suggested that they could have equivalent surgical skills of their category-zone, rather than skills determined by previous surgical experience, detectable by Mocap-based objective skill assessment. As described in Results, the analyzed metrics were roughly grouped into two categories: speed-related and efficiency-related metrics. Among the metrics, Hem-o-lok-related metrics strongly contributed to the axis of the 1st principal component. As shown in Fig. 6, experts better handled a clip applier in the “near zone (2.0  ≤ to < 4.0 cm from aorta) with a shorter idle state and quicker movements, while the analysis of the distribution of the working area revealed that the trajectory of the Hem-o-lok applier was longer in the “near zone (2.0  ≤ to < 4.0 cm from aorta)” in novice and intermediate groups. These observations strongly suggest an autonomous state of experts without wondering of the instrument before determining the closure site on the vascular pedicle.

Limitations of this study include the small sample size and heterogeneity, for example, differences in background (medical students/a junior resident/urologists) and the inclusion of two left-handed surgeons. Experience does not always reflect the level of actual skills and expertise, although 15 of the 18 experts had the ESSQ credential. Mocap data do not necessarily reflect errors and quality outcomes. Regarding intermediate and expert groups, only urological doctors participated in the present study. Although we used 50 cases as a cutoff-point for the definition of the expert group based on our previous validation study of the present model, hundreds of cases may be needed to make one an expert surgeon in real world clinical practice. In order to confirm the generality of our observation and gain further insights into expertise in laparoscopic surgery, we started second data collection, inviting laparoscopic surgeons other than those with urological backgrounds. Nevertheless, we believe that our study contributes to in-depth understanding of surgical dexterity and the process of learning laparoscopic surgical skills in terms of motion metrics. Such knowledge could help educators develop a training curriculum and provide valuable feedback to trainees. Our next challenge involves revising the computer program for metrics calculation and developing an appropriate evaluation form that gives “completely objective” real-time feedback to trainees, and this could become a very powerful educational tool along with experts’ feedback. Furthermore, automatic motion analyses using machine learning is also one of our goals.

Our study is an initial step in a more ambitious research plan to develop a Mocap system that can be utilized in live animal surgery, cadaveric surgical training, or a real clinical setting. This would involve examining the most suitable position of the tracking camera in the operating theater to avoid interruptions in a potentially busy surgical environment. Additionally, easily sterilizable, durable, and light-weight material for the artificial markers used to tag the surgical devices should be sought in a future study [10]. We just started several amelioration including a use of another motion camera system with better portability that does not require calibration, in order to perform Mocap analysis in live animal/cadaveric surgical training as the next stage. Because the present Mocap system can track multiple surgical instruments simultaneously, it provides a novel type of surgical record like a “music score including several musical instruments” during complex procedures, which might become a novel educational tool. We are aiming to develop integrated simulation training using different training models, in conjunction with the feedback of motion parameters to participants.

## Conclusions

Our novel Mocap system identified significant differences in several motion metrics (e.g., path length, velocity, acceleration, and jerk) for multiple surgical instruments during a series of wet-lab training sessions according to the level of surgical experience. “Applying a Hem-o-lok clip on a pedicle” strongly reflected the level of surgical experience, and the exchange of instruments should be included in a training curriculum. Zone-metrics may be a promising tool to asses surgical expertise. Our next challenge is to give completely objective feedback to trainees on-site, which could become a very powerful educational tool along with experts’ feedback.

## Electronic supplementary material

Below is the link to the electronic supplementary material.

Supplementary file1 (DOCX 1012 kb)
