# Correction to: Motion analysis for better understanding of psychomotor skills in laparoscopy: objective assessment-based simulation training using animal organs

**DOI:** 10.1007/s00464-020-08038-w

**Published:** 2020-09-29

**Authors:** Koki Ebina, Takashige Abe, Madoka Higuchi, Jun Furumido, Naoya Iwahara, Masafumi Kon, Kiyohiko Hotta, Shunsuke Komizunai, Yo Kurashima, Hiroshi Kikuchi, Ryuji Matsumoto, Takahiro Osawa, Sachiyo Murai, Teppei Tsujita, Kazuya Sase, Xiaoshuai Chen, Atsushi Konno, Nobuo Shinohara

**Affiliations:** 1 Department of Urology, Hokkaido University Graduate School of Medicine, North-15, West-7, North Ward, Sapporo, 060-8638 Japan; 2 Graduate School of Information Science and Technology, Hokkaido University, Sapporo, Japan; 3 Hokkaido University Clinical Simulation Center, Hokkaido University Graduate School of Medicine, Sapporo, Japan; 4 Department of Mechanical Engineering, National Defense Academy, Yokosuka, 239-8686 Japan; 5 Department of Mechanical Engineering and Intelligent Systems, Tohoku Gakuin University, Tagajo, 985-8537 Japan; 6 Graduate School of Science and Technology, Hirosaki University, Hirosaki, 036-8561 Japan

## Correction to: Surgical Endoscopy 10.1007/s00464-020-07940-7

This article was updated to correct the labeling of Fig. 6.

