# Supplementary material for: Motion analysis for better understanding of psychomotor skills in laparoscopy: objective assessment-based simulation training using animal organs
Source: Surg Endosc. 2020 Sep 9;35(8):4399–416. doi: 10.1007/s00464-020-07940-7 (PMC8263434; doi:10.1007/s00464-020-07940-7)
Supplement: Supplementary file 1 — Supplementary file1 (DOCX 1012 kb) [file 464_2020_7940_MOESM1_ESM.docx]

Supplementary Table 1. Summary of measurements by Mocap system divided by the ESSQ qualification status. (median [interquartile range])
